# Portal vein thrombosis is associated with an increased risk of bone fractures

**DOI:** 10.1371/journal.pone.0267535

**Published:** 2022-04-22

**Authors:** Simon Johannes Gairing, Peter Robert Galle, Jörn Markus Schattenberg, Karel Kostev, Christian Labenz

**Affiliations:** 1 Department of Internal Medicine I, University Medical Center of the Johannes Gutenberg-University, Mainz, Germany; 2 Cirrhosis Center Mainz (CCM), University Medical Center of the Johannes Gutenberg-University, Mainz, Germany; 3 Epidemiology, IQVIA, Frankfurt am Main, Germany; Medizinische Fakultat der RWTH Aachen, GERMANY

## Abstract

**Background:**

Portal vein thrombosis (PVT) is a rare but severe disease that often leads to portal hypertension-related complications. It is well-known that patients with portal hypertension associated with liver cirrhosis are at increased risk for bone fractures, however data on the impact of PVT on fracture risk are lacking.

**Aims:**

This study aimed to explore the impact of PVT on the incidence of bone fractures in a large German primary care cohort.

**Methods:**

Patients with PVT were extensively matched to non-PVT individuals in a 1:5 ratio. The primary outcome of the study was the incidence of any bone fracture.

**Results:**

This study included 596 patients with PVT and 2,980 non-PVT individuals. During five years of follow-up, the cumulative incidence of bone fractures was significantly higher in PVT patients (n = 87, 13.6%) than in those without PVT (n = 186, 6.7%) (p<0.001). In Cox-regression analyses, PVT was positively associated with bone fractures (HR: 2.16; 95% CI: 1.59–2.93). This association was stronger in women (HR: 2.55; 95% CI: 1.65–3.95) than in men (HR: 1.87; 95% CI: 1.22–2.87). The strongest association was observed in the age group 51–60 years (HR: 2.50, 95% CI: 1.40–4.47). The association between PVT and bone fractures was maintained in subgroup analyses of patients with (HR: 2.03, 95% CI: 1.13–3.63) and without liver cirrhosis (HR: 1.82, 95% CI: 1.28–2.58).

**Conclusions:**

PVT is independently associated with a higher incidence of bone fractures. Patients with PVT should be critically evaluated for fracture risk and preventive measures should be considered.

## Introduction

Portal vein thrombosis (PVT) is a severe and, especially if chronic, difficult-to-treat disease entity [1, 2]. PVT is frequently associated with liver cirrhosis, with a prevalence ranging from 1.3–9.8% in cirrhotic patients [1, 3]. Non-cirrhotic PVT is a comparatively rare disease and is driven either by local or systemic prothrombotic conditions such as myeloproliferative diseases [4, 5]. While acute PVT is treated primarily by therapeutic anticoagulation, management of chronic PVT with portal hypertension and cavernous transformation remains a challenge for clinicians, requiring a multidisciplinary approach with individualized therapeutic concepts for the underlying disease and associated complications [1, 6].

Bone fractures represent a frequent complication in patients with liver cirrhosis and portal hypertension. In addition, fractures in patients with liver cirrhosis are associated with an increased 30-days in-hospital mortality [7]. Fractures are often a devastating event in chronically ill patients, and especially hip and vertebral fractures increase the risk of death [8]. In patients with liver cirrhosis-driven portal hypertension fractures are frequently related to incident falls: up to 16% of patients without prior overt hepatic encephalopathy (HE) experience fall-related injuries within 3 years, and incident falls are independently association with increased mortality [9, 10]. Regardless of the underlying etiology, PVT can lead to portal hypertension within a few weeks [1]. Given the fact that liver cirrhosis and PVT share portal hypertension and its consequences as a common complication, it seems reasonable that PVT may be a risk factor for bone fractures on its own. However, there are currently no data on this topic available. Therefore, we hypothesized that PVT might be independently associated with an increased risk of bone fractures.

## Methods

### Database

This study was based on data from the Disease Analyzer database (IQVIA), which compiles drug prescriptions, diagnoses, and basic medical and demographic data obtained directly and in anonymous format from computer systems used in the practices of general practitioners and specialists [11]. Diagnoses (International Classification of Diseases, 10^th^ revision [ICD-10]), prescriptions (European Pharmaceutical Market Research Association [EPhMRA] Anatomical Therapeutic Chemical Classification [ATC] system), and the quality of reported data are monitored by IQVIA based on a number of criteria (e.g., completeness of documentation and linkage between diagnoses and prescriptions). The sampling method for the Disease Analyzer database is based on statistics from all physicians in Germany. These statistics are used to determine the panel composition according to the following strata: region, community size category, and age of physician [11].

### Study population

This study included adult (≥18 years) patients who had an initial diagnosis of PVT (ICD-10: I81) in one of 1,274 general practices Germany between January 2000 and December 2019 (index date). Patients had to have a follow-up time of at least six months after the index date. Patients with osteoporosis (ICD-10: M80, M81), or bone fracture diagnoses (ICD-10: S02, S12, S32, S42, S52, S62, S72, S82, S92, T02, T08, T10, T12) within 12 months prior to or on index date were excluded. After applying similar inclusion criteria, patients without PVT were matched to those with PVT. Greedy nearest-neighbor propensity score matching (1:5) based on sex, age, co-diagnoses documented within 12 months prior to the index date (diabetes mellitus (ICD-10: E10-E14), obesity (ICD-10: C66), liver fibrosis and cirrhosis (ICD10: K70.3, K74) or chronic hepatitis (ICD10: K73), thrombophlebitis (ICD-10: I80), varicose (ICD10: I83-I85)) and yearly consultation frequency was performed. Prior to the matching, these co-diagnoses were either more frequently coded among patients with PVT or were associated with fracture risk in feasibility analyses. Finally, we matched for consultation frequency, as patients with PVT may have a much higher consultation frequency than patients without PVT and this may lead to a higher likelihood of coding a diagnosis.

**Fig 1:**
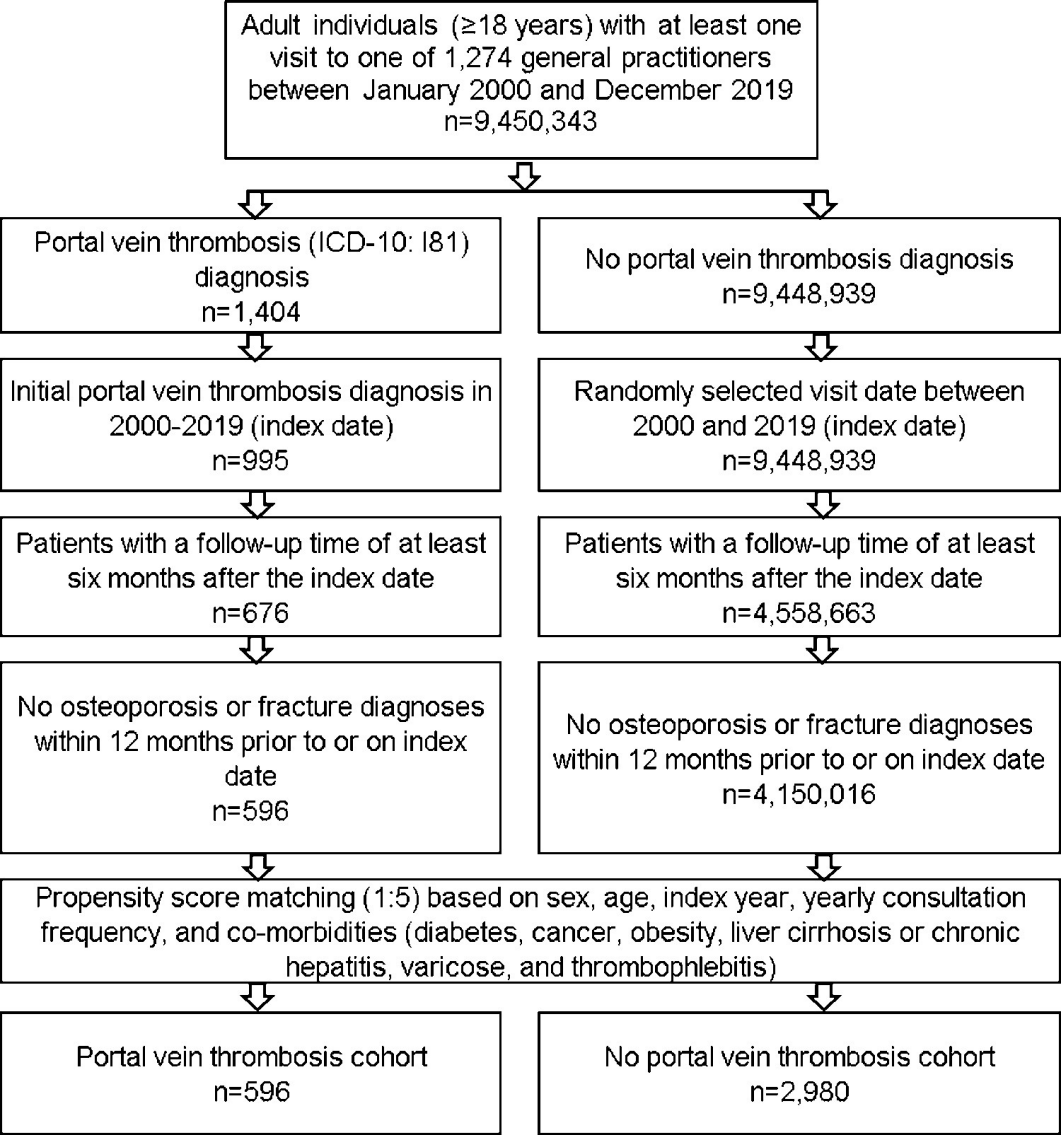
Selection of study patients.

For individuals without PVT, the index date corresponded to a randomly selected visit date between January 2000 and December 2019 (Fig 1). After matching, we additionally compared the frequency of inflammatory bowel diseases (ICD-10: K50, K51) and renal failure (ICD-10: N18, N19) between the groups. Moreover, drugs potentially associated with fracture risk prescribed within 12 months prior to the index date were included as additional variables (corticosteroids, calcium, opioids and sedatives (EphMRA ATC Classes: H02, A12A, N02A, N05B)).

### Ethics

This study was conducted according to the ethical guidelines of the 1964 Declaration of Helsinki (amended, 2013). We used anonymous electronic medical records for research purposes with no directly identifiable data. Accordingly, this study did not collect informed consent from individual patients and according to German regulations no ethical approval is needed. Anonymized data were analyzed as aggregates with no protected health information available.

### Study outcome and statistical analyses

The study outcome was the cumulative incidence of any fracture as function of portal vein thrombosis. After 1:5 matching, the age, sex, co-morbidities, and yearly consultation frequency of portal vein thrombosis patients were compared with those without portal vein thrombosis using McNemar tests for categorical variables and paired Wilcoxon signed-rank test for continuous variables. Kaplan-Meier curves were used to compare the incidence of fractures in the five years following the index year between patients with and without portal vein thrombosis. As there was no information on mortality, dead patients were considered lost to follow-up. Finally, the relationship between portal vein thrombosis, and fracture diagnoses in the overall sample and separately in women and men, four age groups, and patients with and without liver cirrhosis was investigated using Cox regression models. The results of the Cox regression analyses are presented as Hazard Ratios (HRs) with 95% CIs. P-values <0.05 were considered statistically significant. All analyses were performed using SAS 9.4 (SAS Institute, Cary, USA).

## Results

### Baseline characteristics

This study included 596 patients with PVT and 2,980 patients without PVT. After 1:5 matching there were no significant differences in age (57.8 years), sex (43% female), comorbidities, drugs prescribed within 12 months prior to the index date (corticosteroids, calcium, opioids and sedatives), and consultation frequency between the PVT cohort and non-PVT cohort (Table 1). There were no prescriptions of bisphosphonates and only <0.03% of vitamin D. In addition, no differences between baseline variables were detected in the subgroup of patients without liver cirrhosis, when comparing patients with and without portal vein thrombosis (each p > 0.05).

**Table 1:** Baseline characteristics of study patients after 1:5 matching.

Variable	Patients with portal vein thrombosis (N = 596)	Patients without portal vein thrombosis (N = 2,980)	P value
Women	43.0	42.9	0.933
Men	57.0	57.1	
Mean age in years (standard deviation)	57.8 (15.6)	57.8 (15.6)	0.984
Age ≤50 years	30.6	30.8	0.983
Age 51–60 years	23.8	23.1	
Age 61–70 years	21.5	21.8	
Age >70 years	24.1	24.3	
Mean number of consultations per year	6.6 (7.9)	6.1 (7.2)	0.171
Diagnoses documented within 12 months prior to the index date			
Diabetes mellitus	34.3	33.7	0.757
Cancer	19.6	18.9	0.674
Obesity	11.8	10.7	0.381
Liver cirrhosis or chronic hepatitis	21.0	18.1	0.091
Thrombophlebitis	18.5	16.9	0.309
Varicose	30.3	27.8	0.179
Inflammatory bowel diseases	3.0	2.8	0.753
Renal failure	7.9	6.6	0.260
Drugs prescribed within 12 months prior to the index date			
Corticosteroids	3.7	4.3	0.498
Calcium	1.0	1.5	0.349
Opioids	2.9	2.9	0.944
Sedatives	3.9	4.5	0.498

Data are percentages unless otherwise specified.

### Incidence of fractures in patients with and without portal vein thrombosis

**Fig 2:**
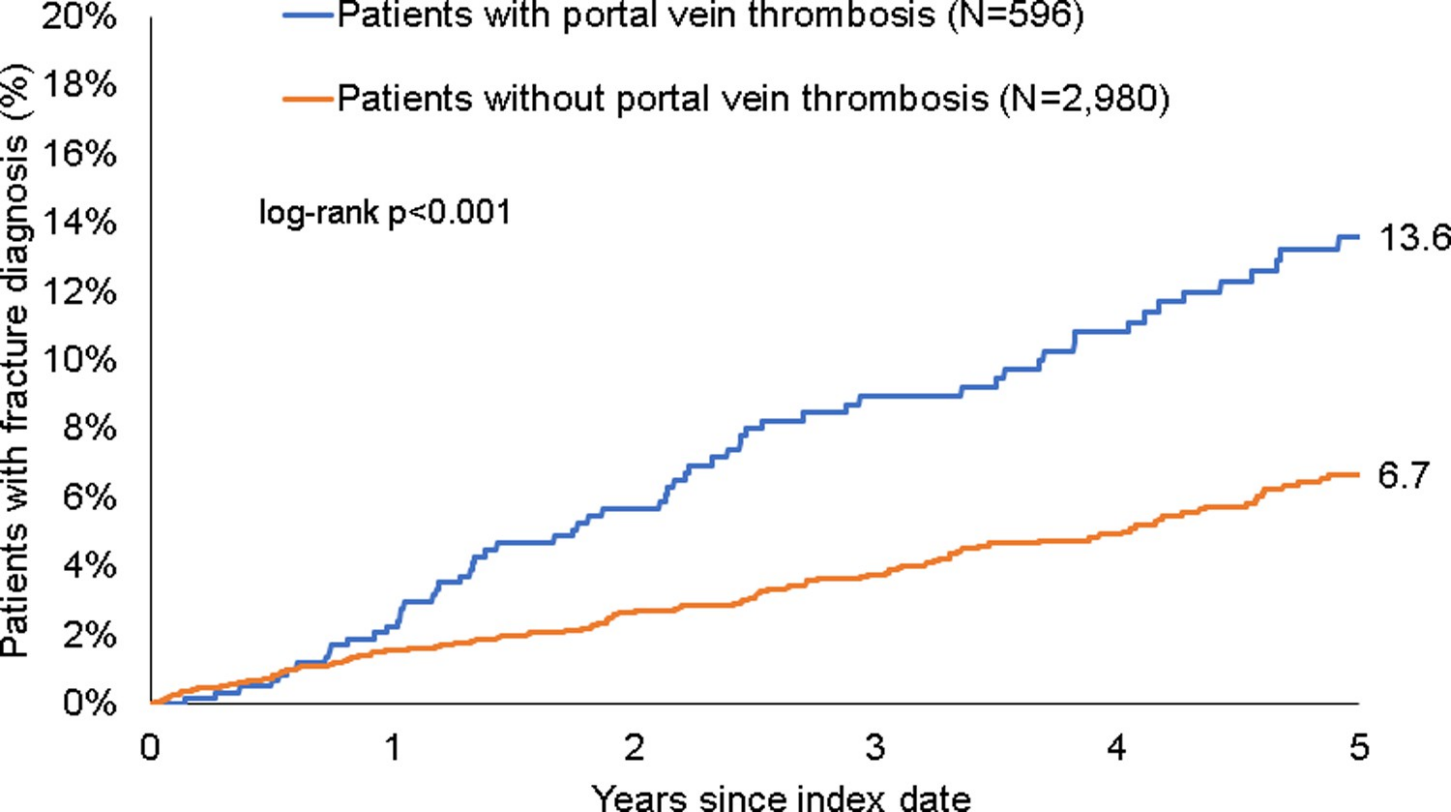
Kaplan-Meier curves for time to a diagnosis of fracture in patients with and without portal vein thrombosis.

A total of 87 patients with PVT and 186 patients without PVT were diagnosed with a bone fracture during the five years of follow-up. The cumulative incidence of bone fractures (13.6% versus 6.7%, log-rank p<0.001) was significantly higher in PVT patients than in those without PVT (Fig 2). The average time between the index date and a bone fracture was 32 months in the PVT cohort and 36 months in the non-PVT cohort. Results of the Cox regression analyses are displayed in Table 2. PVT was positively associated with fractures (HR: 2.16; 95% CI: 1.59–2.93). This association was higher in women (HR: 2.55; 95% CI: 1.65–3.95) than in men (HR: 1.87; 95% CI: 1.22–2.87). The highest association was observed in the age group 51–60 years (HR: 2.50, 95% CI: 1.40–4.47), and the smallest in the age group ≤50 years (HR: 1.86, 95% CI: 0.92–3.83). The latter did not reach statistical significance. The association between PVT and bone fractures remained robust in subgroup analyses of patients with (HR: 2.03, 95% CI: 1.13–3.63) and without liver cirrhosis (HR: 1.82, 95% CI: 1.28–2.58).

**Table 2:** Association between portal vein thrombosis and the 5-year-incidence of fractures (univariable Cox regression analysis).

Cohort	Patients with portal vein thrombosis (Incidence in cases per 1,000 patients years)	Patients without portal vein thrombosis (Incidence in cases per 1,000 patients years)	Hazard Ratio (95% CI)	P value
Overall	28.8	14.1	2.16 (1.59–2.93)	<0.001
Women	34.4	15.6	2.55 (1.65–3.95)	<0.001
Men	24.6	13.1	1.87 (1.22–2.87)	0.004
Age ≤50 years	13.4	8.2	1.86 (0.92–3.83)	0.086
Age 51–60 years	32.2	13.1	2.50 (1.40–4.47)	0.002
Age 61–70 years	39.5	19.9	2.39 (1.36–4.22)	0.003
Age >70 years	48.7	21.2	2.06 (1.12–3.78)	0.020
Patients with liver cirrhosis	49.7	26.0	2.03 (1.13–3.63)	0.017
Patients without liver cirrhosis	25.4	12.6	1.82 (1.28–2.58)	<0.001

### Sites of fractures in patients with and without portal vein thrombosis

**Fig 3:**
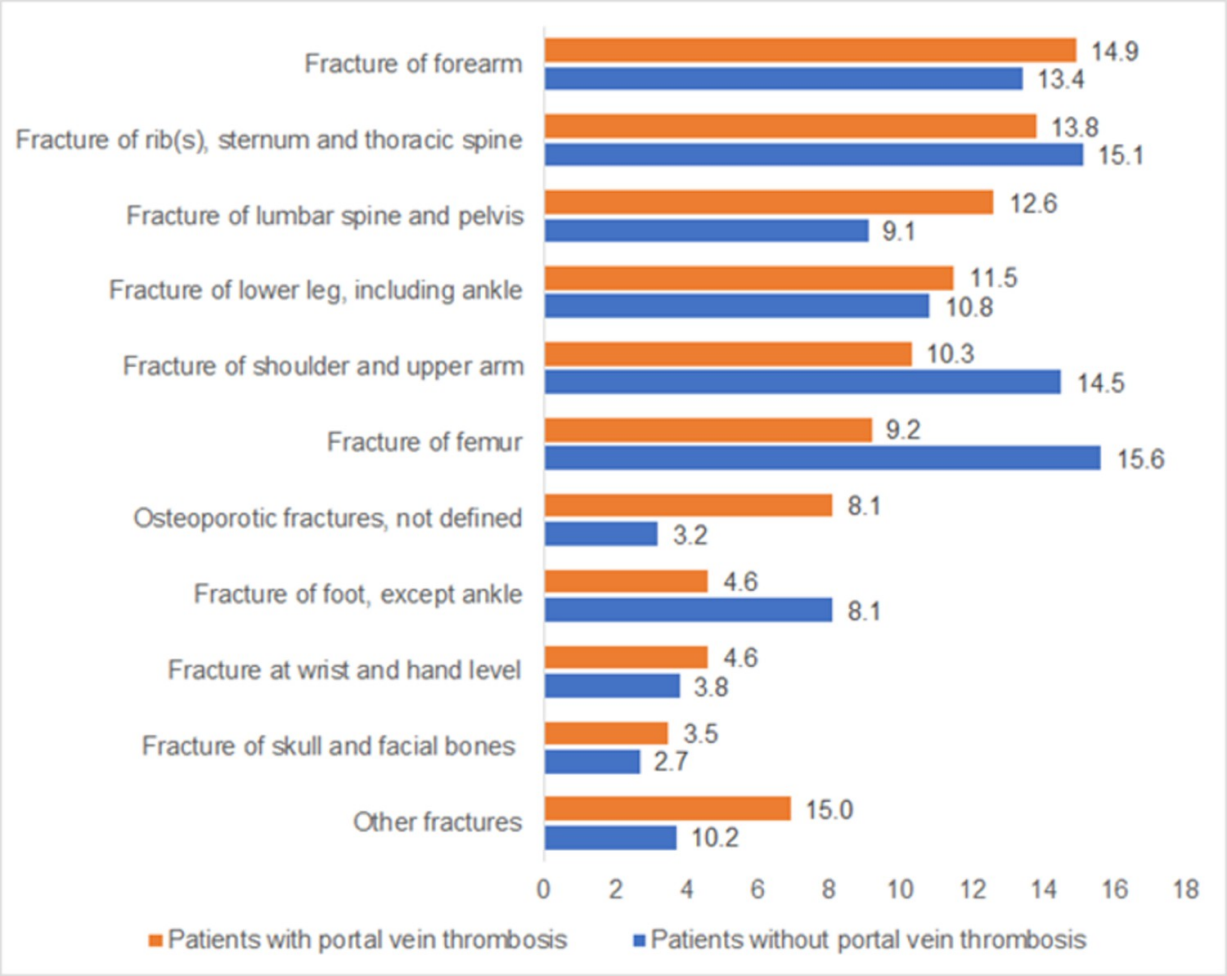
Proportions of different fractures in patients with and without portal vein thrombosis. Data are given as percentages.

Fig 3 shows the proportions of different fractures in patients with and without PVT. The proportions of the most frequent fractures including forearm, rib(s), sternum and thoracic spine, lower leg incl. ancle were similar in patients with and without PVT. In contrast, fractures of shoulder and upper arm, femur, and foot were less frequently observed in patients with PVT than without PVT.

## Discussion

In this study, we demonstrate that the risk of bone fractures is high in patients with PVT. Additionally, we found a relevant and independent positive association between PVT and incident bone fractures. This association was more pronounced in women than in men and remained highly significant across different age groups. Moreover, the association between PVT and bone fractures was independent of the presence of liver cirrhosis.

Bone fractures are associated with both severe morbidity and increased mortality in patients with chronic diseases [12, 13]. In our current study, we found an incidence rate of 28.8 cases with bone fractures per 1,000 person years in patients with PVT, which was 2-fold higher compared to matched individuals without PVT. To the best of our knowledge, our study is the first to investigate the impact of PVT on bone fractures. Due to similarities regarding complications of portal hypertension and its complications, patients with PVT are best comparable to patients with liver cirrhosis. In patients with liver cirrhosis, especially with alcoholic cirrhosis, several studies were able to demonstrate a robust association with bone fractures [14]. A population-based study by Otete et al. using English and Danish databases even found a more than 5-fold increase in the rates of hip fractures when compared to controls [15]. Tsai et al. retrospectively assessed the risk of bone fractures in patients with liver cirrhosis and predominantly hepatitis B and found a 4-fold increase in skull fracture risk in patients with hepatic encephalopathy, while patients without hepatic encephalopathy had an increased risk for spine, trunk und upper limp fractures [16]. Compared to the study by Otete et al., the association found in our study was more moderate with an HR of 2.16, which is most likely explained by the confounding of alcohol abuse in their study [15]. Nevertheless, our study is consistent with these previous reports investigating patients with portal hypertension caused by liver cirrhosis and adds to the existing literature that PVT appears to be an additional independent risk factor for bone fractures irrespective of the presence of liver cirrhosis.

Due to our study design, we can only detect associations and are unable to prove causality or delineate pathophysiological links. However, there are some potential explanations for the strong statistical association between PVT and bone fractures. First, patients with PVT suffer from portal hypertension, which may in turn lead to the development of portosystemic shunts. These may cause minimal hepatic encephalopathy, which is a well-studied cause of falls and serious injuries like bone fractures [9, 10]. In this context, Minguez et al. were able to demonstrate that even patients with non-cirrhotic PVT develop subclinical neurological abnormalities compatible with minimal hepatic encephalopathy [17]. Second, it is well known that patients with cirrhotic as well as non-cirrhotic portal hypertension frequently suffer from sarcopenia [18]. Sarcopenia, in turn, may increase the risk of falls and the occurrence of bone fractures in general. However, the pathophysiological role of portal hypertension for the development of sarcopenia is currently unknown. Taken together, these hypotheses have to be investigated in future well-designed prospective studies.

Currently, the management of patients with (chronic) PVT mainly focusses on the prevention of complications of portal hypertension (such as variceal bleeding or ascites) and the management of the underlying disease. Our study has shown that bone fractures are frequent in these patients and that PVT is an independent and additive risk factor. These findings have the clinical implication that patients with PVT should be critically evaluated for fracture risk and preventive measures should be considered.

To the best of our knowledge, this is the first study to investigate in detail the impact of PVT on bone fracture risk.

The major strengths of our study are the comparatively large patient cohorts, the long follow-up period and the careful matching. However, there are also some limitations that need to be considered when interpreting our data. Due to our study design, we cannot consider minimal HE or sarcopenia in our analyses, as these are not coded in the Disease Analyzer Database. The same is true for chronic alcohol abuse, which can affect the risk of falls and therefore fractures. Additionally, vitamin D was only prescribed in < 0.03% of our patients. This is most likely explained by the fact that vitamin D is an over the counter drug in Germany and we are therefore unable to adjust for this variable in our analyses. Taken together, only prospective randomization, in contrast to retrospective matching, can control unknown, potentially confounding baseline characteristics. Only 21% of patients with PVT in our cohort had a concurrent coded diagnosis of liver cirrhosis. This may be explained by undercoding of PVT in patients with liver cirrhosis treated in primary care. However, the size of our cirrhosis cohort was sufficiently powered to detect an association between PVT and fractures in patients with liver cirrhosis. In our cohort, no patient had a code for hematological malignancies or prothrombotic diseases. It seems likely that these patients were coded as “cancer” in this primary care database, however, this has to be acknowledged as a limitation of our study. Last, the Disease Analyzer database does not capture detailed laboratory values nor information on disease stages. Therefore, we cannot investigate the effect of different grades of portal hypertension or PVT on the incidence of bone fractures.

In conclusion, we found that patients with PVT are at high risk for bone fractures. Additionally, we observed a robust association of PVT with the incidence of bone fractures when compared to individuals without PVT. Future prospective studies are needed to validate our findings. Additionally, strategies to identify patients with PVT at high risk of bone fractures should be intensified to implement preventive interventions.

## Abbreviations

PVT: Portal vein thrombosis
HR: hazard ratio
CI: confidence intervall
HE: hepatic encephalopathy
IQVIA: Disease Analyzer database
ICD-10: International Classification of Diseases, 10th revision
EPhMRA: European Pharmaceutical Market Research Association
ATC: Anatomical Therapeutic Chemical Classification
